# EVALUATION OF AN ALZHEIMER’S DISEASE TRAINING FOR LATINX COMMUNITY HEALTH WORKERS

**DOI:** 10.1093/geroni/igae098.0468

**Published:** 2024-12-31

**Authors:** Sonia Bishop, Kelley Pascoe, Janet Rojina, Katherine Lopez, Theresa Kehne, Karen Torres, Kimiko Domoto-Reilly, Linda Ko

**Affiliations:** University of Washington, Seattle, Washington, United States; University of Washington, Seattle, Washington, United States; University of Washington, Seattle, Washington, United States; University of Washington, Seattle, Washington, United States; University of Washington, Seattle, Washington, United States; University of Washington Harborview Medical Center, Seattle, Washington, United States; University of Washington, Seattle, Washington, United States; University of Washington, Seattle, Washington, United States

## Abstract

Despite an anticipated 800% increase in Alzheimer’s disease (AD) prevalence among U.S. Hispanic/Latinx by 2060, Hispanic/Latinx are persistently underrepresented in AD research, which is partly due to low participation in Alzheimer’s Disease Research Centers (ADRC). Community Health Workers (CHWs) are trusted community members and can serve as educators of AD and ADRC. This study evaluated changes in pre and post knowledge about AD and ADRC among CHWs serving Hispanic/Latinx after delivery of a culturally and linguistically appropriate AD training. The training covered AD prevalence and risk factors, AD preventive behaviors, knowledge about ADRC, and the importance of AD research. Pre/post assessment was conducted using non-matched, anonymous surveys; differences in pre/post measures (mean+SD) were estimated and analyzed using Wilcoxon-Mann-Whitney U tests. Participants (N=68) had a mean (SD) age of 40 (10.7) years, were majority female (87%), identified as Latinx (91%), born outside the U.S. (55%) and represented 21 Washington counties. There was a significant increase in knowledge of AD (pre: 2.16 + 1.25; post: 3.51 + 1.09; p< 0.001), AD signs and symptoms (pre: 2.04 + 1.25, post: 3.38 + 1.13; p< 0.001), and AD prevention (pre: 1.84 + 1.16, post: 3.46 + 1.19; p< 0.001) pre/post training. Similarly, there was significant increase in knowledge of the local ADRC (pre: 1.38 + 0.92, post: 3.27 + 1.48; p< 0.001), ADRC studies (pre: 1.63 + 1.11, post: 3.31 + 1.22; p< 0.001) and what ADRC participation entails (pre: 2.59 + 1.74, post: 4 + 1.17; p< 0.001) pre/post training. Culturally and linguistically concordant training for CHWs showed promise for increasing knowledge about AD and ADRC. Future research should explore how CHWs use training information to educate Hispanic/Latinx individuals about AD and ADRC.

